# Fast Bayesian inference for gene regulatory networks using ScanBMA

**DOI:** 10.1186/1752-0509-8-47

**Published:** 2014-04-17

**Authors:** William Chad Young, Adrian E Raftery, Ka Yee Yeung

**Affiliations:** 1 Department of Statistics, Univesity of Washington, 98195, Seattle WA, USA; 2 Department of Microbiology, University of Washington, Box 357735, 98195-7735, Seattle WA, USA

**Keywords:** Bayesian inference, Bayesian model averaging, Gene regulatory networks

## Abstract

**Background:**

Genome-wide time-series data provide a rich set of information for discovering gene regulatory relationships. As genome-wide data for mammalian systems are being generated, it is critical to develop network inference methods that can handle tens of thousands of genes efficiently, provide a systematic framework for the integration of multiple data sources, and yield robust, accurate and compact gene-to-gene relationships.

**Results:**

We developed and applied ScanBMA, a Bayesian inference method that incorporates external information to improve the accuracy of the inferred network. In particular, we developed a new strategy to efficiently search the model space, applied data transformations to reduce the effect of spurious relationships, and adopted the *g*-prior to guide the search for candidate regulators. Our method is highly computationally efficient, thus addressing the scalability issue with network inference. The method is implemented as the ScanBMA function in the networkBMA Bioconductor software package.

**Conclusions:**

We compared ScanBMA to other popular methods using time series yeast data as well as time-series simulated data from the DREAM competition. We found that ScanBMA produced more compact networks with a greater proportion of true positives than the competing methods. Specifically, ScanBMA generally produced more favorable areas under the Receiver-Operating Characteristic and Precision-Recall curves than other regression-based methods and mutual-information based methods. In addition, ScanBMA is competitive with other network inference methods in terms of running time.

## Background

Identifying gene regulatory networks is an important problem in biology. There have recently been many advances in this area in terms of tools for collecting and analyzing large-scale genomics data. Many of these datasets, from microarrays and next generation sequencing, quantify the expression levels of all genes in a given genome. Genome-wide time-series data, in principle, allow reverse engineering of the gene regulatory relationships by studying the temporal patterns of regulators and target genes. However, this can be a difficult problem due to the large number of genes (*i.e.* variables) being measured, which typically far exceeds the number of observations. Also, the number of actual regulators for a particular gene is only a small fraction of the number of possible regulators.

A popular method for inferring gene regulatory networks from time series data uses Dynamic Bayesian Networks (DBN) [1-5]. DBN methods estimate a probabilistic graphical model, given the time-series data. DBN methods work well, but the network size that they can handle in practice is limited because of their computational cost.

Ordinary differential equations (ODE) are alternative methods for constructing networks [6,7]. These methods are deterministic rather than statistical, although ODE methods can be combined with statistical methods. DBN on local networks within a larger ODE model inference method have been used, for example [8].

Another class of methods is based on regression models in which parent nodes (regulators) are inferred for each target gene. Vector autoregressive models have been proposed for inferring causal links between genes. Often this takes the form of a model selection problem, and methods such as the Least Absolute Shrinkage and Selection Operator (LASSO) [9,10], elastic net [11,12], and Bayesian model averaging (BMA) [13,14] have been used [15-19]. Morrissey, et al. [20] implemented a Markov Chain Monte Carlo (MCMC) sampler for a fully Bayesian formulation of the autoregressive model.

Mutual information methods have been used extensively on genetics data [21-24], but usually for steady-state rather than time-series data. These methods are typically non-directional. Recently, mutual information methods have been extended to analyze time-series data and produce directed networks [25,26]. Mutual information methods have the advantage of being able to identify nonlinear relationships.

### Our contributions

We present a new approach using Bayesian Model Averaging (BMA) for variable selection from time-series gene expression data. Our new method offers the following advances over our previous work [18,19]: 

• We develop a new algorithm called ScanBMA that searches the model space more efficiently and thoroughly than previous algorithms. It is much faster than previous implementations of BMA for a large number of predictors, resulting in running time comparable to that of LASSO. It allows inference for networks of thousands of genes to be completed in minutes on a standard laptop computer.

• We transform the time-series data to reduce spurious correlations. Specifically, we remove the effect of a gene on itself by subtracting the mean expression level for each gene at each time point and then using the residuals from a regression of its expression at the current time point on its expression at the previous time point.

• We use Zellner’s *g*-prior [27] for the regression parameters and show that using the *g*-prior to compute posterior probabilities out-performs our previous effort using the Bayesian Information Criterion (BIC).

• We address the scalability of network inference methods. Our new implementation produces running times of minutes compared to hours or even days for some competing methods, thus offering substantial improvements.

We also carried out extensive empirical studies of our new method. Specifically, we compared our new method, ScanBMA, to other network construction methods in the literature, namely LASSO, the mutual information methods MRNET (Maximum Relevance/Minimum Redundancy) [24], CLR (Context Likelihood or Relatedness) [23] and ARACNE (Algorithm for the Reconstruction of Accurate Cellular Networks) [22], and also Dynamic Bayesian Networks (DBN) when the latter were computationally feasible.

We benchmarked the performance of our approach, ScanBMA, using two datasets. The first dataset measures the gene expression levels over time of 97 yeast segregants perturbed with the drug rapamycin. The second dataset consists of simulated time-series data from the DREAM4 (Dialogue for Reverse Engineering Assessment and Methods) challenge. For the yeast dataset, we found that our method outperformed competitors and previous analyses in recovering regulatory relationships previously reported in the literature. For the DREAM4 data, for which no prior information was available, our method performed comparably to other methods, while producing more compact networks. Finally, the ScanBMA algorithm presents a substantial improvement in running time over previous implementations of BMA. The method is implemented as the ScanBMA function in the networkBMA Bioconductor software package.

## Results and discussion

### Method outline

In ScanBMA, network inference is formulated as a series of variable selection problems in which parent nodes (regulators) are inferred for each target gene. The BMA framework accounts for model uncertainty in variable selection by averaging over the posterior distributions from multiple models, weighted by their posterior model probabilities [13,14]. A challenge of BMA is to efficiently select a set of models to be averaged over. ScanBMA uses a greedy approach to explore the model space and uses the Occam’s window principle [28] to eliminate unlikely models.

We previously developed a supervised framework to integrate external data sources, including co-expression, genome-wide binding, sequence polymorphism, physical interaction, genetic interaction, and literature curation data [18]. Using a training set consisting of approximately 500 known regulatory relationships in the literature, we computed prior probabilities of regulatory relationships across all candidate genes and regulators. These prior probabilities were then used to compute the posterior probabilities of of candidate regression models. We used Zellner’s *g*-prior [27] to specify the prior for the model parameters in ScanBMA. We developed an expectation-maximization (EM) algorithm to estimate the prior variance parameter *g*.

Before the regression step, we apply a univariate measure (such as R-squared or BIC) to rank candidate regulators for each target gene using these prior probabilities of regulatory relationships. The parameter *nvar* controls the number of top regulators used in the regression step of each target gene. We have performed empirical studies to study the effect of and estimate the optimal *nvar*.

### Assessment

A number of metrics have been used to evaluate the quality of inferred networks. We focus on a few that compare the inferred network with a gold-standard network of true edges. One measure that we use is the precision of the inferred network, equal to the number of true positives divided by the total number of edges in the inferred network. Precision is important to researchers because an experiment to verify relationships identified in exploratory work can be expensive. Thus, the more confident we can be when identifying relationships, the better. In light of this, we also look at the area under the precision-recall curve (AUPRC). This gives a more comprehensive view of network quality and does not require that a threshold be chosen for the posterior probability of an edge or for the number of edges included. We also look at the area under the ROC curve (AUROC), which is widely used to assess the quality of networks.

Due to incomplete knowledge in real data, we use a partial assessment based on the YEASTRACT database [29]. This is a literature-curated repository of regulatory relationships between known transcription factors and target genes in yeast, based on more than 1,300 literature references. Due to incomplete knowledge in yeast biology, the lack of an edge in YEASTRACT is not hard evidence of the absence of a relationship between two genes, although it is used as such in our evaluation. In contrast, the true underlying networks from the simulated DREAM4 data are known, so that an absence of a true edge is a false negative and a presence of a non-existing edge is a false positive.

### Results: yeast time series data

Table 1 summarizes the assessment results for different methods applied to the yeast dataset. Our new ScanBMA method with *nvar* = 20 had a precision of 0.39, much higher than any other method, including our previous method, iBMA [19]. The area under the ROC curve (AUROC) for ScanBMA was also much better than for the competing methods. Note that for random guessing, the expected AUROC is 0.5 and the expected AUPRC is 0.038. For these data, the mutual information methods CLR and MRNET produced very large networks — nearly half of the number of possible edges, which is not concordant with what is known about regulatory networks of this type.

**Table 1:** Performance of different methods on the yeast data

**Method**	**Precision**	**AUROC**	**AUPRC**	**TP**	**FP**
LASSO	0.046	0.506	0.0416	996	20,469
ARACNE	0.205	0.502	0.0399	69	268
CLR	0.039	0.510	0.0435	8,879	220,942
MRNET	0.039	0.513	0.0442	8,737	214,757
ScanBMA ^[20]^	**0.391**	0.601	0.0747	227	353
ScanBMA ^[3556]^	0.274	**0.629**	0.0740	127	336
iBMA ^[100]^	0.180	0.517	**0.0788**	593	2,702

AUROC is the area under the ROC curve, AUPRC is the area under the precision-recall curve, and TP and FP are the numbers of true positive and false positive edges inferred, respectively. Thus TP +FP is the number of edges in the inferred network and Precision = TP/(TP +FP). ScanBMA was applied to the transformed data using the informative edge prior and Zellner’s *g*-prior for the model parameters. The superscript indicates the value of *nvar*. Expected precision and AUPRC from random guessing is 0.0380.

Table 2 compares the preferred version of ScanBMA with four other versions, each of which lacks one of the components of our final method. This table shows that each component contributes to the accuracy of our final network. As shown by [19], the incorporation of the informative prior yields the largest increase in performance, but the other components also contribute. The use of the *g*-prior reduces the number of false positives, while the data transformation substantially reduces the size of the inferred network.

**Table 2:** Performance of different versions of ScanBMA on the yeast data, either on the original scale (Orig) or transformed (Trans)

**Data**	**nvar**	**Priors**	**Precision**	**AUROC**	**AUPRC**	**TP**	**FP**
Trans	20	*g*, inform	**0.391**	0.601	**0.0747**	227	353
Trans	3556	*g*, inform	0.274	**0.629**	0.0740	127	336
Trans	20	BIC, inform	0.244	0.590	0.0616	200	619
Orig	20	*g*, inform	0.274	0.586	0.0680	552	1460
Trans	20	*g*, Guelzim	0.175	0.499	0.0395	34	160

For the priors, inform refers to the informative prior, while Guelzim refers to the prior probability of 2.76/6000 for all possible relationships.

**Figure 1:**
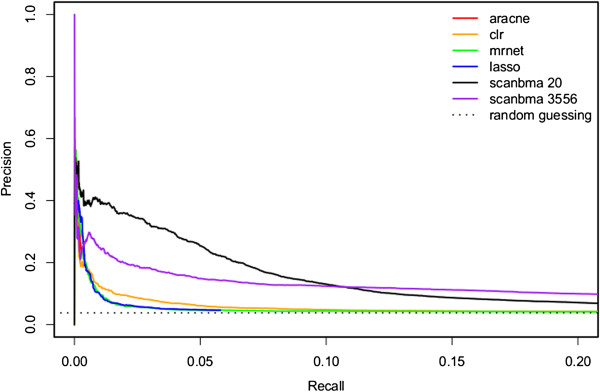
**Yeast precision-recall curves.** Precision-Recall curves for different methods on the yeast data. ScanBMA was run using the *g*-prior, transformed data, and informative prior with nvar = 20 and 3556.

The precision-recall curves for the different methods on the yeast data are shown in Figure 1. This figure shows the quality of the ScanBMA network even beyond the 95% posterior inclusion probability cutoff. The precision stays high through a large range of recall, whereas for the other methods it quickly drops to the level of random guessing. The figure also shows that *nvar* = 20 performs better than *nvar* = 3556.

When analyzing gene networks where the number of nodes is in the thousands, computation time can be an important consideration. We compared ScanBMA with the other methods on the yeast data by running each method on 20 target genes under controlled conditions to find the average cpu time per gene.

Table 3 shows that ScanBMA with *nvar* = 3556, i.e. considering all other genes whose expression varied as potential regulators, is within a factor of 3 of LASSO, the fastest of the other methods. Some of the mutual information methods, on the other hand, are much slower, with MRNET taking about 50 times longer than ScanBMA. Table 3 also shows that ScanBMA produces a substantial improvement in computational efficiency over our previous method iBMA [19], especially when *nvar* is large. Dynamic Bayesian network methods were not included in the comparison because they analyze the entire network at once and do not scale well enough to be run feasibly on large network datasets such as the yeast data.

**Table 3:** Average CPU time per target gene of different methods for the yeast data

**Method**	**Running Time Per Gene (s)**
LASSO	4.1
ARACNE	70.4
CLR	7.9
MRNET	>500
ScanBMA ^[20]^	0.04
ScanBMA ^[3556]^	11.2
iBMA ^[20]^	0.08
iBMA ^[3556]^	85

ScanBMA was run with *g*-prior, transformed data, informative prior. Superscript indicates value of *nvar*.

### Results: simulated DREAM4 data

Table 4 summarizes the results of the competing methods for the DREAM4 10-gene networks. For the DREAM4 networks, we were able to add the Dynamic Bayesian Networks method, as implemented in the ebdbnet R package [30], to the comparison because the networks are small. ScanBMA again performed best among the methods, particularly in terms of the areas under the ROC and precision-recall curves, even though no external information was available. However, the extent of ScanBMA’s superiority to other methods, notably LASSO, was smaller in this case than for the yeast data, reflecting the lack of external information.

**Table 4:** Average performance of different methods on the DREAM4 10-gene networks

**Method**	**Precision**	**AUROC**	**AUPRC**	**TP**	**FP**
LASSO	0.190	0.731	0.487	62	265
ebdbnet	**0.509**	0.704	0.438	28	27
ARACNE	0.304	0.668	0.388	35	80
CLR	0.215	0.681	0.397	50	183
MRNET	0.215	0.709	0.409	53	193
ScanBMA	0.432	**0.740**	**0.505**	35	46

ScanBMA was run with the original data. The true positive (TP) and false positive (FP) columns are totaled across all 5 networks. There are 71 true edges across the 5 networks.

**Figure 2:**
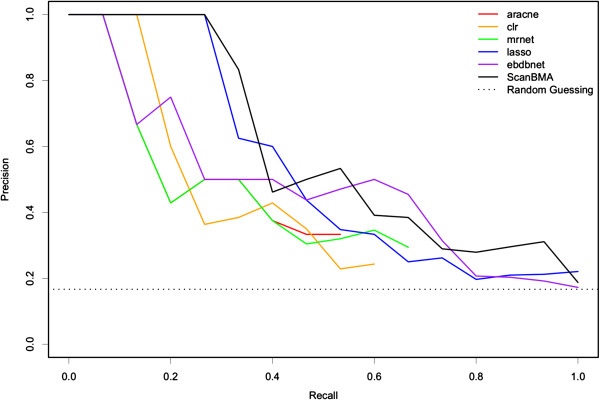
**10-gene Precision-Recall curves.** Precision-Recall curves for various methods on network 1 of the 10-gene networks from the DREAM4 competition. This network has 15 true edges.

**Figure 3:**
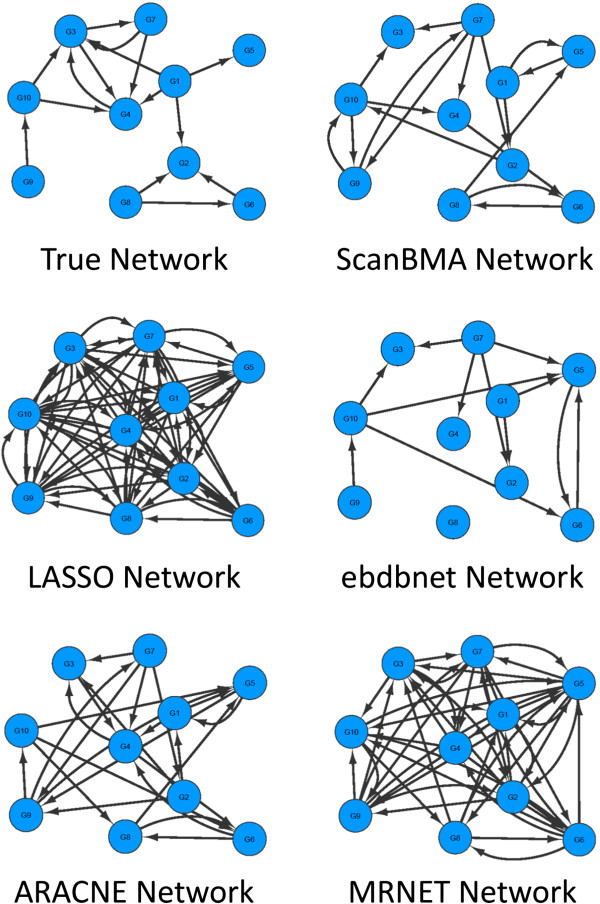
DREAM4 10-gene network visual comparison.

The precision-recall curves from the various methods are shown for the first of the five 10-gene networks in Figure 2. Figure 3 shows the actual networks returned by each method in comparison with the true network. The ScanBMA network resembles the true network fairly well. In particular, the compactness of the ScanBMA network is apparent, particularly when compared with LASSO and MRNET. The small number of false positives may be useful in focusing the attention of the biologist on edges of high interest when searching for new regulatory relationships.

**Figure 4:**
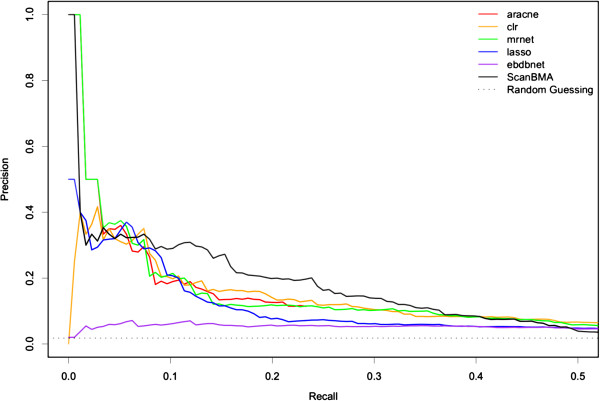
**100-gene Precision-Recall curves.** Precision-Recall curves for various methods on network 1 of the 100-gene networks from the DREAM4 competition.

The results of the methods for the DREAM4 100-gene networks are summarized in Table 5. For these networks, the mutual information methods MRNET and CLR performed best in terms of the area under curve measures. ScanBMA was not quite as good by these measures, but its precision was much higher than that of any other method. Figure 4 illustrates the precision-recall curves for the various methods, showing increased precision across a broader range for ScanBMA.

**Table 5:** Average performance of different methods on the DREAM4 100-gene networks

**Method**	**Precision**	**AUROC**	**AUPRC**	**TP**	**FP**
LASSO	0.035	0.643	0.073	571	15757
ebdbnet	0.054	0.643	0.043	182	3201
ARACNE	0.114	0.589	0.106	208	1621
CLR	0.035	0.699	0.123	678	18669
MRNET	0.035	**0.701**	**0.130**	689	18784
ScanBMA	**0.153**	0.657	0.101	193	1062

ScanBMA run with original data. The true positive (TP) and false positive (FP) columns are totaled across all 5 networks. There are 1,024 true edges across the 5 networks

## Conclusions

We have presented a Bayesian Model Averaging method for inferring gene regulatory networks from time series data. It incorporates external information in a principled way via the prior edge probabilities, transforms the data to reduce spurious correlations, and uses Zellner’s *g*-prior for model parameters, with *g* estimated from the data. We have introduced a new algorithm, ScanBMA, to search the model space efficiently. Our method infers compact networks with higher precision than the competing methods we have assessed, important features for further analysis in searching for new regulatory relationships.

We found that our method outperformed previous methods as well as LASSO and mutual information methods on yeast time-series data. In addition, our method performed comparably to competing methods, including Dynamic Bayesian Networks, on simulated data from the DREAM4 challenge, even in the absence of prior information. The networks from ScanBMA are also similar in size to the target networks.

## Methods

### Bayesian model averaging

In regression-based methods for network inference, we infer regulators (parent nodes) for each target gene. Hence, network inference can be formulated as a series of variable selection problems. We use the following multiple linear regression model for network inference from time-series data:

$$X_{i,t,s}=\beta_{0,i}+\underset{h\in H}{\sum }\beta_{\text{hi}}X_{h,t-1,s}+\varepsilon_{i,t,s},$$

where *X*_*i*,*t*,*s*_ is the expression level for gene *i* at time *t* for strain or replicate *s*, *H* is the set of potential regulators, and $\varepsilon_{i,t,s}\overset{\text{iid}}{∼}N(0,\sigma_{\varepsilon }^{2})$ is the error term, for *i* = 1,…,*n*, *t* = 2,…,*T* and *s* = 1,…,*S*. We are particularly interested in whether *β*_*hi*_ ≠ 0, indicating a regulatory relationship from gene *h* to gene *i*.

One difficulty is that for gene network time series data there are typically far more potential regulators than observations. To address this problem, as well as to take into account uncertainty in model selection, we use BMA to obtain the posterior probability that each regulator is in the model [13,14]. BMA takes model uncertainty into account by averaging over the posterior distributions of a quantity of interest based on multiple models, weighted by their posterior model probabilities. Thus the posterior probability that *β*_*hi*_ ≠ 0, also called the posterior inclusion probability of *β*_*hi*_, is

$$\text{Pr}(\beta_{\text{hi}}\neq 0|D)=\overset{K}{\underset{k=1}{\sum }}1(\beta_{\text{hi}}\neq 0|D,M_{k})\text{Pr}(M_{k}|D),$$

where $\text{Pr}(M_{k}|D)\propto \int p(D|\theta_{k},M_{k})p(\theta_{k}|M_{k})d\theta_{k}$ is the posterior probability of model *M*_*k*_ given data *D*, *p*(*D*|*θ*_*k*_,*M*_*k*_) is the likelihood of the parameter vector *θ*_*k*_ of model *M*_*k*_, the prior probability of model *M*_*k*_ is $\text{Pr}(M_{k})\propto \prod_{h\in M_{k}}\pi_{\text{hi}}\prod_{h\notin M_{k}}(1-\pi_{\text{hi}})$, *π*_*hi*_ is the prior probability that gene *h* regulates gene *i*, and the models considered are denoted by *M*_1_,…,*M*_*K*_.

An additional issue is that the number of possible models is too large to enumerate in a reasonable amount of time. MCMC approaches have been applied where the number of potential regulators is large [31], but they are expensive computationally. Iterative BMA has also been applied to large genetics datasets, but the number of regulators was restricted prior to analysis [18,32].

### Data transformations

One concern when identifying regulatory relationships among genes is that there is a great deal of variation in gene expression levels that does not come from these interactions. For example, in the yeast data, many of the genes experience a sharp change in expression level over the first few time points caused by the application of the drug. This common trajectory is not important for inference and can produce many large correlations that do not correspond to actual interactions. In addition, we have found that removing the effect of a gene on itself can improve inference. By doing this we are removing excess variation in order to gain accuracy in inferring relationships.

In light of these observations, we transform the data in two steps to remove these extra sources of variation. First, we use time-adjusted data by subtracting the mean expression level for each gene *i* at timepoint *t* across strains:

$$X_{i,t,s}^{*}=X_{i,t,s}-{\bar{X}}_{i,t,\cdot },$$

where ${\bar{X}}_{i,t,\cdot }=S^{-1}\sum_{s=1}^{S}X_{i,t,s}$. This removes the overall effect of the drug.

Second, we take the residuals from regressing the gene on itself at the previous timepoint:

$$X_{i,t,s}^{*}=X_{i,t,s}-{\hat{\alpha }}_{i}X_{i,t-1,s},$$

where *α*_*i*_ is the regression coefficient in the simple linear regression model of the expression level of gene *i* at time *t* on its expression level at time *t* - 1:

$$X_{i,t,s}=\alpha_{i}X_{i,t-1,s}+\delta_{i,t,s},$$

where $\delta_{i,t,s}\overset{\text{iid}}{∼}N(0,\sigma_{i}^{2})$. Then ${\hat{\alpha }}_{i}$ is the least squares estimate of *α*_*i*_ based on all *S*(*T* - 1) relevant observations for gene *i*.

### Specifying prior distributions

An important feature of our method is a way to incorporate external information. The Bayesian approach requires the specification of prior distributions and so includes this directly.

BMA requires two types of prior information: the prior edge probability, *π*_*hi*_, that potential regulator *h* regulates target gene *i*, for each *h* and *i*, and the prior distribution of the parameter vector for each model considered.

For the prior edge probabilities *π*_*hi*_, we considered two different prior distributions. The first is based on the empirical finding of Guelzim et al. [33] that each target gene is regulated by a small number of transcription factors, estimated as 2.76 per gene [19]. We implement this by setting *π*_*hi*_ = 2.76/6000 for all *h* and *i*. This prior distribution does not incorporate any gene-specific information. We call it the Guelzim prior.

The second prior edge distribution we consider is that of Lo et al. [19], which uses external information in the form of expression data, binding-site data, curated literature and other sources to come up with a prior probability for each individual possible regulatory relationship. This leads to values of *π*_*hi*_ specific to each regulator-target pair. We refer to this as the informative prior. Integration of multiple information sources has been shown to be beneficial in network construction [34,35].

As a prior for the model parameters, corresponding to the strengths of the relationships, we use Zellner’s *g*-prior [27], as in [36]. The prior distribution of the parameter vector $\left(\beta_{0,i}^{\left[k\right]},\beta^{\left[k\right]},\sigma^{2[k]}\right)$ of model *M*_*k*_ is

$$\begin{array}{l} \;\beta^{\left[k\right]}|\sigma^{2[k]},M_{k},g∼\;N(0,g\sigma^{2[k]}{\left(X_{k}^{T}X_{k}\right)}^{-1}),\\ p(\beta_{0,i}^{\left[k\right]},\sigma^{2[k]}|M_{k})\propto \;1/\sigma^{2[k]}, \end{array}$$

where *X*_*k*_ is the design matrix for model *M*_*k*_ and *g* > 0 controls the prior variance of the regression parameters. This prior yields an analytic form for the posterior model probabilities *Pr*(*M*_*k*_|*D*), namely

$$\begin{array}{l} 2\log\left(p(M_{k}|D)/p(M_{0}|D)\right)\\ =(n-1)\log(1+g(1-R_{k}^{2}))-(n-d_{k}-1)\log(1+g)\\ \;-2\underset{h\in M_{k}}{\sum }\;\log\;\left(\frac{\pi_{\text{hi}}}{1-\pi_{\text{hi}}}\right), \end{array}$$

where *M*_0_ is the null model with no regulators, *d*_*k*_ is the number of regulators present in model *M*_*k*_, and $R_{k}^{2}$ is the *R*^2^ value for *M*_*k*_. This is an alternative to using BIC to approximate the posterior model probabilities, as has been done previously [32,37].

The parameter *g* controls the expected size of the regression parameters *β*_*hi*_, and is approximately equal to the prior variance of $\beta_{\text{hi}}/\text{SE}({\hat{\beta }}_{\text{hi}})$, where ${\hat{\beta }}_{\text{hi}}$ is the OLS estimator. This suggests that *g* should be at least 1, otherwise the individual *β*_*hi*_’s would be expected to be nearly indistinguishable from the noise even under ideal conditions. Also, the effective number of data points in the prior is *n*/*g*. Using *g* = *n* corresponds to a unit information prior and yields similar results to using the BIC approximation. Raftery [38] argued that the prior should be no more spread out than the unit information prior, suggesting a choice of *g* in the range 1 ≤ *g* ≤ *n*.

We estimate *g* from the data by maximum marginal likelihood, where the likelihood is summed over the model space. We do this using an EM algorithm [39,40], where the “missing data” are the *γ*_*hi*_’s indicating whether regulator gene *h* is in the model for target gene *i*. First, we run BMA with a selected value of *g*, and from this we obtain the posterior probabilities of the models used. We then maximize

$$\begin{array}{l} \overset{K}{\underset{k=1}{\sum }}\text{Pr}(M_{k}|D)\left(\left(n-d_{k}-1)\log(1+g\right)\right)\\ \;\left(-(n-1)\log\left[1+g(1+R_{k}^{2})\right]\;\right) \end{array}$$

with respect to *g*. We use the new value of *g* in the next iteration of ScanBMA. We do this until convergence.

### Searching the model space using ScanBMA

To find the models to be included in the BMA summation, we use the Occam’s window principle, according to which models that are substantially worse than the best model found (by a factor of *C* in posterior probability) are discarded [28]. We used *C* = 100, based on an extensive review of conventional standards of scientific evidence. To find the models in Occam’s window, we propose a new, fast algorithm called ScanBMA.

The main idea of ScanBMA is to keep an active set of models around which to search. All models which can be created by adding or removing a single predictor from those in the active set are then evaluated and, if they fall within Occam’s window of the current best model, are added to the active set to expand the search. The method is outlined in Algorithm 1.

**Algorithm 1:** ScanBMA

Because ScanBMA does not average over every model in the model space, the algorithm yields posterior inclusion probabilities of either 100% or 0% for many regulators. These extreme posterior inclusion probabilities are only approximations, and we can refine them. To estimate the posterior inclusion probability of a predictor *X*_*h*_ with an approximate posterior probability of 100%, we calculate the ratio *O*_-*h*,*i*_ of the posterior probability of the best model to that of the best model with the predictor *X*_*h*_ removed. We then approximate the posterior probability of predictor *h* by *O*_-*h*,*i*_/(1 + *O*_-*h*,*i*_), which will be less than 100%.

Similarly, to estimate the posterior inclusion probability of a predictor *X*_*h*_ with approximate posterior probability 0%, we compute *O*_*h*,*i*_, the ratio of the posterior probability of the best model to that of the best model with the predictor *X*_*h*_ added. Then the approximate posterior inclusion probability of predictor *h* is (1 + *O*_*h*,*i*_)^-1^, which will be greater than 0%.

This post-processing step yields a unique ordering among the predictors, which is useful in evaluation.

A variant of ScanBMA that we found to greatly improve computational efficiency without degrading performance is to restrict the number, *nvar*, of potential regulators *h* considered for each target gene *i* to those with the highest prior probabilities, *π*_*hi*_. Guelzim et al. [33] found that the number of transcription factors per gene has approximately an exponential distribution with mean 2.76; they did not find any of the target genes that they considered to have more than 13 regulators. As a result, we consider *nvar* = 20, which leaves some margin over the Guelzim maximum. We compare this with considering all genes with observed variation in expression as potential regulators, which amounts to setting *nvar* = 3556.

### Data

To validate our method, we applied it to time-series data from a gene expression experiment on yeast as well as simulated time-series datasets from the DREAM4 competition.

The yeast data come from an experiment on 97 strains of yeast crossed from two parent strains [18]. Each strain was subjected to a treatment of the macrolide drug rapamycin, chosen to cause changes in gene transcription across the genome. Gene expression levels were measured for approximately 6,000 genes every 10 minutes from 0 to 50 minutes after the administration of the drug, using Affymetrix microarrays. The data were then filtered to remove genes that did not show significant variation over the measurements, leaving 3,556 genes.

The yeast data can be represented as a three-dimensional array consisting of 3,556 genes, 97 segregants and 6 time points. There are no replicates in these data. However, the segregant axis and the time axis capture the genetic and temporal variations. These yeast data are publicly available from ArrayExpress with accession number E-MTAB-412.

The simulated DREAM4 In Silico Network Challenge [41-43] provided 5 networks each of size 10 and 100 genes [44]. Time-series data for each network are produced by artificially perturbing a portion of the genes in the network and simulating the response of the network over time.

Specifically, the size 10 DREAM data consist of 10 genes, 21 time points and 5 replicates. The size 100 DREAM data consist of 100 genes, 21 time points, and 10 replicates.

Since our focus is on time-series data, we did not use the other data sources provided by the DREAM4 challenge. In particular, the DREAM4 challenge provided the results of simulated gene knock-out experiments for all genes, and in fact the winning entry in the competition used only the knock-out data and ignored the time series data [45]. In practice, however, this is unrealistic, since it is typically feasible to do knock-out experiments for only a small proportion of the genes. Time series data do have the potential to provide some information about all potential regulatory relationships, and our goal is to develop methods for doing this, so we have ignored the knock-out data here.

### Competing methods

To evaluate the performance of our method, we compared it with LASSO [9], as well as with the mutual information methods MRNET, CLR and ARACNE. LASSO is a variable selection method based on a linear regression model, and thus can be used as an alternative to BMA. It is known for being computationally efficient.

We used the implementation of LASSO in the glmnet package in R[12], with the shrinkage penalty parameter, sometimes denoted by *λ*, chosen by cross-validation.

Mutual information methods have been used extensively in identifying relationships among genes [21-23]. Since mutual information methods are non-directional, we used a modified version inspired by [46], including the response as the first column of the matrix given to the mutual information method, and the predictors as the other columns. We then took the first column from the resulting mutual information matrix as the measure of the directed relationship from the predictors to the target gene. MRNET, CLR and ARACNE are different methods for using the mutual information to infer a weighted adjacency matrix between genes. All are implemented in the minet package in R[47].

We have also investigated the possibility of using Dynamic Bayesian Networks (DBN). Methods based on DBNs have been implemented in several R packages. These include the GeneNet package [48], but this is not designed for multiple time-series and so was not a comparable method on our datasets. The ebdbnet (Empirical Bayes Estimation of Dynamic Bayesian Networks) R package [30], and the G1DBN R package [49], do allow for multiple time-series, but they are not designed to handle networks of the size of the yeast data. They are more appropriate for networks of sizes up to a few hundred genes. We were able to use the ebdbnet package for the much smaller DREAM4 networks.

### YEASTRACT: Assessment of yeast data

The YEASTRACT database [29] is a literature-curated repository of regulatory relationships between known transcription factors and target genes in yeast, based on more than 1,300 literature references. Among the 3,556 genes in our filtered yeast time-series data, this database contains documented regulatory relationships for 127 transcription factors (TFs), encompassing a total of 17,173 edges. We therefore evaluate only inferred relationships from these 127 transcription factors, and inferred regulatory relationships from other genes are not used in evaluation.

## Availability of supporting data

The yeast time series data are publicly available from ArrayExpress http://www.ebi.ac.uk/arrayexpress[50] with accession number E-MTAB-412. The DREAM4 data are publicly available from http://wiki.c2b2.columbia.edu/dream/index.php?title=D4c2[44].

## Abbreviations

ARACNE: Algorithm for the reconstruction of accurate cellular networks; BMA: Bayesian model averaging; CLR: Context likelihood or relatedness; DBN: Dynamic Bayesian network; DREAM: Dialogue for reverse engineering assessments and methods; LASSO: Least absolute shrinkage and selection operator; MRNET: Maximum relevance/minimum redundancy; PRC: Precision recall curve; ROC: Receiver operating characteristic; TF: Transcription factor; YEASTRACT: Yeast search for transcriptional regulators And consensus tracking.

## Competing interests

The authors declare that they have no competing interests.

## Authors’ contributions

WCY participated in method development, implemented the ScanBMA method, carried out empirical studies comparing ScanBMA to other competing methods, and drafted the manuscript. AER designed the ScanBMA algorithm, designed the empirical studies and edited the manuscript. KYY identified the datasets, participated in the design of the empirical studies, and assisted in manuscript preparation. All authors read and approved the final manuscript.

## References

[B1] Murphy K Mian S Modelling gene expression data using dynamic Bayesian networks Technical report, Computer Science Division, University of California, Berkeley, CA, 1999

[B2] Kim S.Y Imoto S Miyano S Inferring gene networks from time series microarray data using dynamic Bayesian networks Brief Bioinform 2003 4 3 228 235 10.1093/bib/4.3.228 14582517

[B3] Kim S.Y Imoto S Miyano S Dynamic Bayesian network and nonparametric regression for nonlinear modeling of gene networks from time series gene expression data Biosystems 2004 75 1 57 65 15245804 10.1016/j.biosystems.2004.03.004

[B4] Zou M Conzen SD A new dynamic Bayesian network (DBN) approach for identifying gene regulatory networks from time course microarray data Bioinformatics 2005 21 1 71 79 10.1093/bioinformatics/bth463 15308537

[B5] Zhu J Chen Y Leonardson AS Wang K Lamb JR Emilsson V Schadt EE Characterizing dynamic changes in the human blood transcriptional network PLOS Comput Biol 2010 6 2 1000671 10.1371/journal.pcbi.1000671 PMC2820517 20168994

[B6] D’haeseleer P Wen X Fuhrman S Somogyi R Linear modeling of mRNA expression levels during CNS development and injury Pacific Symposium on Biocomputing (PSB) conference: January 4-9, 1999; Hawaii, Volume 4 1999 41 52 10.1142/9789814447300_0005 10380184

[B7] Bansal M Della Gatta G Di Bernardo D Inference of gene regulatory networks and compound mode of action from time course gene expression profiles Bioinformatics 2006 22 7 815 822 10.1093/bioinformatics/btl003 16418235

[B8] Li Z Li P Krishnan A Liu J Large-scale dynamic gene regulatory network inference combining differential equation models with local dynamic bayesian network analysis Bioinformatics 2011 27 19 2686 2691 10.1093/bioinformatics/btr454 21816876

[B9] Tibshirani R Regression shrinkage and selection via the lasso J R Stat Soc Series B (Methodol) 1996 58 267 288

[B10] Tibshirani R Saunders M Rosset S Zhu J Knight K Sparsity and smoothness via the fused lasso J R Stat Soci: Series B (Stat Methodol) 2005 67 1 91 108 10.1111/j.1467-9868.2005.00490.x

[B11] Zou H Hastie T Regularization and variable selection via the elastic net J R Stat Soc: Series B (Stat Methodol) 2005 67 2 301 320 10.1111/j.1467-9868.2005.00503.x

[B12] Friedman J Hastie T Tibshirani R Regularization paths for generalized linear models via coordinate descent J Stat Softw 2010 33 1 1 22 20808728 PMC2929880

[B13] Raftery AE Madigan D Hoeting JA Bayesian model averaging for linear regression models J Am Stat Assoc 1997 92 437 179 191 10.1080/01621459.1997.10473615

[B14] Hoeting JA Madigan D Raftery AE Volinsky CT Bayesian model averaging: a tutorial Stat Sci 1999 14 382 401 10.1214/ss/1009212519

[B15] Gustafsson M Hörnquist M Lundström J Björkegren J Tegnér J Reverse engineering of gene networks with LASSO and nonlinear basis functions Ann N Y Acad Sci 2009 1158 1 265 275 10.1111/j.1749-6632.2008.03764.x 19348648

[B16] Menéndez P Kourmpetis Y ter Braak C van Eeuwijk FA Gene regulatory networks from multifactorial perturbations using Graphical Lasso: application to the DREAM4 challenge PLOS ONE 2010 5 12 14147 10.1371/journal.pone.0014147 PMC3004794 21188141

[B17] Shojaie A Michailidis G Discovering graphical Granger causality using the truncating lasso penalty Bioinformatics 2010 26 18 517 523 10.1093/bioinformatics/btq377 PMC2935442 20823316

[B18] Yeung KY Dombek KM Lo K Mittler JE Zhu J Schadt EE Bumgarner RE Raftery AE Construction of regulatory networks using expression time-series data of a genotyped population Proc Nat Acad Sci 2011 108 48 19436 19441 10.1073/pnas.1116442108 22084118 PMC3228453

[B19] Lo K Raftery A Dombek K Zhu J Schadt E Bumgarner R Yeung KY Integrating external biological knowledge in the construction of regulatory networks from time-series expression data BMC Syst Biol 2012 6 1 101 10.1186/1752-0509-6-101 22898396 PMC3465231

[B20] Morrissey ER Juárez MA Denby KJ Burroughs NJ On reverse engineering of gene interaction networks using time course data with repeated measurements Bioinformatics 2010 26 18 2305 2312 10.1093/bioinformatics/btq421 20639410

[B21] Basso K Margolin AA Stolovitzky G Klein U Dalla-Favera R Califano A Reverse engineering of regulatory networks in human b cells Nat Genet 2005 37 4 382 390 10.1038/ng1532 15778709

[B22] Margolin AA Nemenman I Basso K Wiggins C Stolovitzky G Favera RD Califano A ARACNE: an algorithm for the reconstruction of gene regulatory networks in a mammalian cellular context BMC Bioinformatics 2006 7 Suppl 1 7 10.1186/1471-2105-7-S1-S7 16723010 PMC1810318

[B23] Faith JJ Hayete B Thaden JT Mogno I Wierzbowski J Cottarel G Kasif S Collins JJ Gardner TS Large-scale mapping and validation of Escherichia coli transcriptional regulation from a compendium of expression profiles PLOS Biology 2007 5 1 8 10.1371/journal.pbio.0050008 PMC1764438 17214507

[B24] Meyer PE Kontos K Lafitte F Bontempi G Information-theoretic inference of large transcriptional regulatory networks EURASIP J Bioinform Syst Biol 2007 2007 79879 18354736 10.1155/2007/79879 PMC3171353

[B25] Zoppoli P Morganella S Ceccarelli M Timedelay-ARACNE: Reverse engineering of gene networks from time-course data by an information theoretic approach BMC Bioinformatics 2010 11 1 154 10.1186/1471-2105-11-154 20338053 PMC2862045

[B26] Lopes F de Oliveira E Cesar R Inference of gene regulatory networks from time series by Tsallis entropy BMC Syst Biol 2011 5 1 61 10.1186/1752-0509-5-61 21545720 PMC3117729

[B27] Zellner A On assessing prior distributions and Bayesian regression analysis with g-prior distributions Bayesian Inference Decis Tech: Essays Honor of Bruno De Finetti 1986 6 233 243

[B28] Madigan D Raftery AE Model selection and accounting for model uncertainty in graphical models using Occam’s window J Am Stat Assoc 1994 89 428 1535 1546 10.1080/01621459.1994.10476894

[B29] Teixeira MC Monteiro P Jain P Tenreiro S Fernandes AR Mira NP Alenquer M Freitas AT Oliveira AL Sá-Correia I The YEASTRACT database: a tool for the analysis of transcription regulatory associations in saccharomyces cerevisiae Nucleic Acids Res 2006 34 suppl 1 446 451 10.1093/nar/gkj013 PMC1347376 16381908

[B30] Rau A Jaffrézic F Foulley J Doerge RW An empirical Bayesian method for estimating biological networks from temporal microarray data Stat Appl Genet Mol Biol 2010 9 1 1544 6115 10.2202/1544-6115.1513 20196759

[B31] Bottolo L Richardson S Evolutionary stochastic search for Bayesian model exploration Bayesian Anal 2010 5 3 583 618 10.1214/10-BA523

[B32] Yeung KY Bumgarner RE Raftery AE Bayesian model averaging: development of an improved multi-class, gene selection and classification tool for microarray data Bioinformatics 2005 21 10 2394 2402 10.1093/bioinformatics/bti319 15713736

[B33] Guelzim N Bottani S Bourgine P Képès F Topological and causal structure of the yeast transcriptional regulatory network Nat Genet 2002 31 1 60 63 10.1038/ng873 11967534

[B34] Zhu J Zhang B Smith EN Drees B Brem RB Kruglyak L Bumgarner RE Schadt EE Integrating large-scale functional genomic data to dissect the complexity of yeast regulatory networks Nat Genet 2008 40 7 854 861 10.1038/ng.167 18552845 PMC2573859

[B35] Yip KY Alexander RP Yan K Gerstein M Improved reconstruction of in silico gene regulatory networks by integrating knockout and perturbation data PLOS ONE 2010 5 1 8121 10.1371/journal.pone.0008121 PMC2811182 20126643

[B36] Clyde M George E. I Model uncertainty Stat Sci 2004 19 81 94 10.1214/088342304000000035

[B37] Raftery AE Bayesian model selection in social research Sociol Methodol 1995 25 111 164

[B38] Raftery AE Bayes factors and BIC Sociol Methods Res 1999 27 3 411 417 10.1177/0049124199027003005

[B39] Dempster AP Laird NM Rubin DB Maximum likelihood from incomplete data via the EM algorithm J R Stat Soc Series B (Methodological) 1977 39 1 1 38

[B40] McLachlan G Krishnan T The EM Algorithm and Extensions. Volume 382 2007 Hoboken, New Jersey: John Wiley & Sons

[B41] Marbach D Prill RJ Schaffter T Mattiussi C Floreano D Stolovitzky G Revealing strengths and weaknesses of methods for gene network inference Proc Nat Acad Sci 2010 107 14 6286 6291 10.1073/pnas.0913357107 20308593 PMC2851985

[B42] Marbach D Schaffter T Mattiussi C Floreano D Generating realistic in silico gene networks for performance assessment of reverse engineering methods J Comput Biol 2009 16 2 229 239 10.1089/cmb.2008.09TT 19183003

[B43] Prill RJ Marbach D Saez-Rodriguez J Sorger PK Alexopoulos LG Xue X Clarke ND Altan-Bonnet G Stolovitzky G Towards a rigorous assessment of systems biology models: the DREAM3 challenges PLOS ONE 2010 5 2 9202 10.1371/journal.pone.0009202 PMC2826397 20186320

[B44] DREAM4 In Silico Network Challenge [ http://wiki.c2b2.columbia.edu/dream/index.php?title=D4c2]

[B45] Pinna A Soranzo N de la Fuente A From knockouts to networks: establishing direct cause-effect relationships through graph analysis PLOS ONE 2010 5 10 12912 10.1371/journal.pone.0012912 PMC2952592 20949005

[B46] Shimamura T Imoto S Yamaguchi R Fujita A Nagasaki M Miyano S Recursive regularization for inferring gene networks from time-course gene expression profiles BMC Syst Biol 2009 3 1 41 10.1186/1752-0509-3-41 19386091 PMC2686685

[B47] Meyer PE Lafitte F Bontempi G minet: AR/Bioconductor package for inferring large transcriptional networks using mutual information BMC Bioinformatics 2008 9 1 461 10.1186/1471-2105-9-461 18959772 PMC2630331

[B48] Schäfer J Opgen-Rhein R Strimmer K Reverse engineering genetic networks using the Genenet package J Am Stat Assoc 2001 96 1151 1160 10.1198/016214501753382129

[B49] Smith SM Fulton DC Chia T Thorneycroft D Chapple A Dunstan H Hylton C Zeeman SC Smith AM Diurnal changes in the transcriptome encoding enzymes of starch metabolism provide evidence for both transcriptional and posttranscriptional regulation of starch metabolism in Arabidopsis leaves Plant Physiol 2004 136 1 2687 2699 10.1104/pp.104.044347 15347792 PMC523333

[B50] ArrayExpress [ http://www.ebi.ac.uk/arrayexpress]

